# Inhibition of nuclear factor-κB signaling suppresses *Spint1*-deletion-induced tumor susceptibility in the *Apc*^Min/+^ model

**DOI:** 10.18632/oncotarget.11863

**Published:** 2016-09-06

**Authors:** Makiko Kawaguchi, Koji Yamamoto, Ai Kanemaru, Hiroyuki Tanaka, Kazuo Umezawa, Tsuyoshi Fukushima, Hiroaki Kataoka

**Affiliations:** ^1^ Section of Oncopathology and Regenerative Biology, Department of Pathology, Faculty of Medicine, University of Miyazaki, Miyazaki, Japan; ^2^ Department of Molecular Target Medicine Screening, Aichi Medical University School of Medicine, Aichi, Japan

**Keywords:** HAI-1, Spint1, DHMEQ, NF-κB, colon cancer

## Abstract

Hepatocyte growth factor activator inhibitor type 1 (HAI-1), encoded by the *Spint1* gene, is a membrane-bound serine protease inhibitor expressed on the epithelial cell surface. We have previously reported that the intestine-specific *Spint1*-deleted *Apc*^Min/+^ mice showed accelerated formation of intestinal tumors. In this study, we focused on the role of nuclear factor-κB (NF-κB) signaling in the HAI-1 loss-induced tumor susceptibility. In the HAI-1-deficient intestine, inflammatory cytokines, such as tumor necrosis factor-α and interleukin-6, were upregulated in normal mucosa. Furthermore, increased nuclear translocation of NF-κB was observed in both normal mucosa and tumor tissues of HAI-1-deficient *Apc*^Min/+^ intestines, and an NF-κB target gene, such as urokinase-type plasminogen activator, was upregulated in the HAI-1-deficient tumor tissues. Thus, we investigated the effect of dehydroxymethylepoxyquinomicin (DHMEQ), a synthetic inhibitor of NF-κB, on intestinal HAI-1-deficient *Apc*^Min/+^ mice. Treatment with DHMEQ reduced the formation of intestinal tumors compared with vehicle control in the HAI-1-deficient *Apc*^Min/+^ mice. These results suggested that insufficient HAI-1 function promotes intestinal carcinogenesis by activating NF-κB signaling.

## INTRODUCTION

Chronic inflammation plays an important role in cancer development [1]. In particular, colorectal carcinoma (CRC) is strongly associated with an inflammatory microenvironment and used as a model of the link between chronic inflammation and cancer. Many studies have shown that inflammatory responses promote intestinal carcinogenesis by altering the intestinal microenvironment [2]. Indeed, experimental colitis enhances intestinal carcinogenesis in *Apc*^Min/+^ mice through an inducible nitric oxide synthase (iNOS)-dependent mechanism [3]. As *Apc* gene mutations are responsible for the familial adenomatous polyposis, and more than 80% of CRC harbor mutations in *APC*, the *Apc*^Min/+^ mice model is considered a relevant intestinal carcinogenesis model [4–5].

Nuclear factor (NF)-κB is a family of transcription factors that regulate the expression of genes involved in cell growth and survival, stress response, and inflammation [6]. For example, urokinase-type plasminogen activator (uPA); vascular endothelial growth factor; and some of the pro-inflammatory factors, such as interleukin-1 (IL-1), tumor necrosis factor (TNF)-α, and iNOS, are regulated by NF-κB [7]. In mammals, NF-κB is composed of RelA (p65), RelB, c-Rel, NF-κB1 (p105/P50), and NF-κB2 (p100/P52). These proteins form homo- or heterodimers. Among them, RelA is the most important subunit of the NF-κB family. Recently, it has become clear that NF-κB signaling plays important roles in the development and progression of cancer [8–10]. NF-κB controls the ability of both preneoplastic and malignant cells to resist apoptosis. It may also regulate tumor angiogenesis and invasiveness. Regarding the effect of NF-κB signaling in colon cancer, significant evidence suggests that NF-κB signaling plays a role in colorectal cancer promotion [2, 11]. Moreover, the activation of NF-κB is reported in over 50% of CRCs [12], suggesting that NF-κB signaling may be a promising therapeutic target in CRCs. Dehydroxymethylepoxyquinomicin (DHMEQ) is a synthetic small molecule that inhibits nuclear translocation of RelA. Previous studies showed that DHMEQ inhibits various types of inflammation and cancer cell growth [13–26].

HAI-1 is a membrane-associated Kunitz-type serine protease inhibitor that is expressed on most epithelial cells and placental cytotrophoblasts [27]. We have previously reported that intestinal barrier function was decreased in intestine-specific HAI-1-deficient mice, and these mice showed increased susceptibility of DSS-induced colitis [28]. Moreover, the loss of HAI-1 accelerated tumor formation both in an inflammation-associated mice colon carcinogenesis model and in a mutant Apc model [29]. However, the molecular mechanism of increased tumor formation in HAI-1-deficient mice is still unclear. Because it was reported that mucosal damage plays a role in the pathogenesis of colitis and CRC [12], we speculated that in the HAI-1-deficient intestine, the inflammatory response was induced and NF-κB signaling was activated, which involved HAI-1 loss-induced acceleration of tumor formation.

In this study, we analyzed the effect of DHMEQ administration on intestinal tumor formation to clarify whether the acceleration of intestinal carcinogenesis observed in HAI-1-deficient mice is associated with the activation of NF-κB signaling or not. We found that NF-κB was activated in HAI-1-deficient *Apc*^Min/+^ mice tumors. Moreover, inhibition of NF-κB by DHMEQ treatment significantly reduced tumor formation in HAI-1-deficient *Apc*^Min/+^ mice. These results suggest that HAI-1 has a suppressive role in intestinal carcinogenesis by regulating of NF-κB signaling.

## RESULTS

### NF-κB signaling was activated in HAI-1-deleted intestine

**Figure 1:**
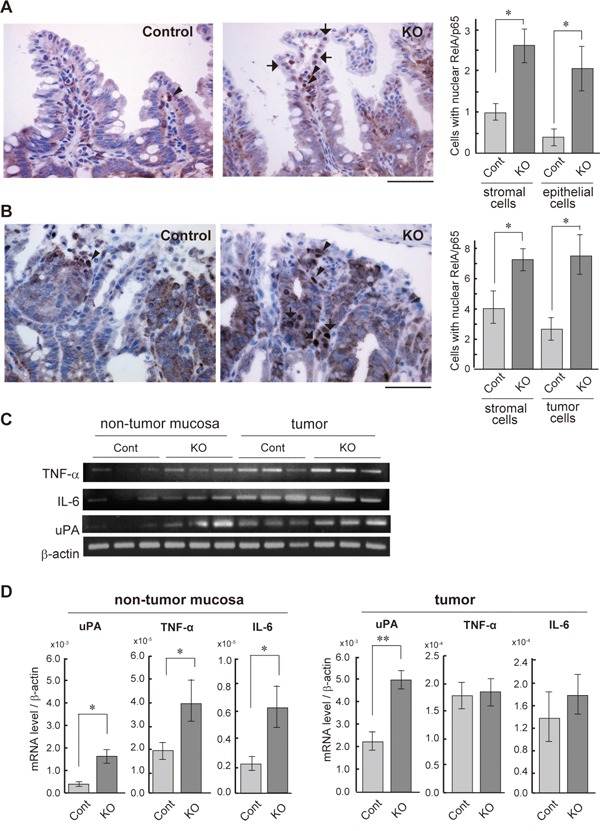
NF-κB activation status of the HAI-1-deficient intestine **(A)** Immunohistochemical analyses of RelA/p65 in the non-tumor mucosa of small intestine from control (Cont; n = 4) and HAI-1-deficient (KO; n = 4) *Apc*^Min/+^ mice at 15 weeks of age. Arrows and arrowheads indicate the nuclear RelA/p65-positive epithelial cells and stromal cells, respectively. Positive cells/200× field were counted and plotted in the right panel (mean ± SEM). *, *p* < 0.05. Bar, 50 μm. **(B)** Nuclear immunoreactivity of RelA/p65 in intestinal tumor tissue. Arrows and arrowheads indicate the nuclear RelA/p65-positive tumor cells and stromal cells, respectively. Right panel indicates mean number ± SEM of positive cells/200× field. *, *p* < 0.05. Bar, 50 μm. **(C)** RT-PCR of NF-κB target genes in non-tumor mucosa and tumor tissues of control and KO mice. **(D)** Quantitative RT-PCR for mRNAs of NF-κB target genes. *, *p* < 0.05, **, *p* < 0.005.

We have previously reported that the loss of HAI-1 in intestinal epithelial cells altered intestinal barrier function and increased susceptibility to experimental colitis [28]. Therefore, we hypothesized that NF-κB signaling is activated in the HAI-1-deficient intestine. To confirm this hypothesis, we evaluated the nuclear translocation of RelA/p65, the subunit of NF-κB, using *Spint1*^LoxP/LoxP^/Villin-Cre^+/0^/ *Apc*^Min/+^ mice (hereafter HAI-1-deficient *Apc*^Min/+^ mice) at 15 weeks of age, the same mice as in our previous study [29]. The defect of HAI-1 protein was confirmed by immunohistochemistry (Supplementary Figure S1). In normal mucosa of the small intestine, an increased number of nuclear RelA/p65-positive cells were observed in both epithelial and stromal components of HAI-1-deficient *Apc*^Min/+^ mucosa compared to control *Apc*^Min/+^ mucosa (Figure 1A). In tumor tissues, the nuclear translocation of RelA/p65 was also increased in both tumor cells and stromal cells of the HAI-1-deficient intestine (Figure 1B). We also examined the expression of NF-κB target genes, such as TNF-α, IL-6, and uPA. In tumor tissue, the expression of TNF-α and IL-6 was increased both in control mice and HAI-1-deficient mice. However, in the non-tumor mucosa, the expression levels of these genes were significantly increased in HAI-1-deficient mice compared with control mice (Figure 1C, 1D). The uPA expression was upregulated both in non-tumor mucosa and tumor tissue in HAI-1 deficient mice (Figure 1C, 1D).

### NF-κB inhibitor alleviated the HAI-1 loss-induced enhanced formation of intestinal tumors

**Figure 2:**
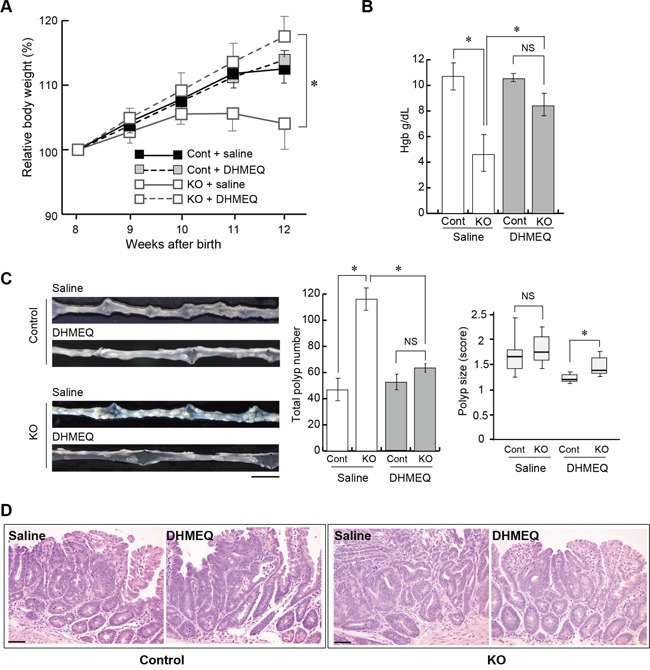
Effect of DHMEQ on intestinal tumorigenesis in HAI-1-deficient *Apc*^Min/+^ mice **(A)** Relative body weight (mean ± SEM) of Cont (saline; n = 6), Cont (DHMEQ; n = 5), KO (saline; n = 5), and KO (DHMEQ; n = 6) groups. Mean body weight at the start point (8 weeks of age) of each group was 21.47 (Cont + saline), 21.42 (Cont + DHMEQ), 21.18 (KO + saline) or 20.33 (KO + DHMEQ), without statistically significant differences between groups. *, p < 0.05. **(B)** Mean hemoglobin concentration (mean ± SEM) of the mice. NS, not significant; *, *p* < 0.05. **(C)** Representative macroscopic appearance of the small intestine from saline- or DHMEQ-treated control and KO mice (left panel), the number of intestinal polyps (mean ± SEM) of the mice (middle panel), and polyp size scores of the mice (right panel). The box shows the interquartile range, and the whiskers indicate the sample maximum and minimum. The median is indicated by a bold line. *, *p* < 0.05. Bar, 1 cm. **(D)** Histology of tumors (H&E stain). Bar, 50 μm.

**Figure 3:**
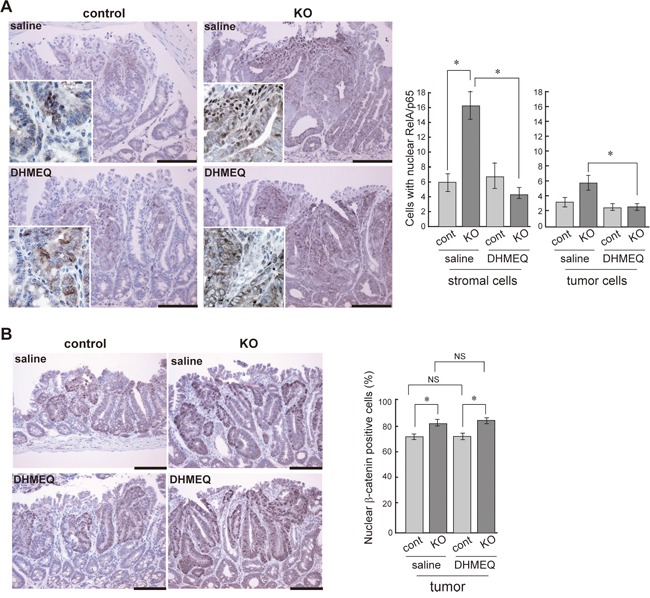
Effect of DHMEQ on nuclear translocation of RelA/p65 and β-catenin in HAI-1-deficient *Apc*^Min/+^ mice tumor Immunostaining of RelA/p65 **(A)**, and β-catenin **(B)** of control and KO tumor before and after DHMEQ treatment. The enlarged image is also shown as an inset. Bar, 100 μm. *, *p* < 0.01. NS, not significant.

We then investigated the effect of DHMEQ on tumor formation in HAI-1-deficient *Apc*^Min/+^ mice. The mice were treated with an intraperitoneal administration of saline with or without DHMEQ (three times per week for 4 weeks) and sacrificed 12 weeks after birth. Ten weeks after birth, the body weight gain of HAI-1-deficient *Apc*^Min/+^ mice was lower than control mice. However, the intraperitoneal administration of DHMEQ alleviated body weight loss in HAI-1-deficient *Apc*^Min/+^ mice (Figure 2A). It is well known that *Apc*^Min/+^ mice exhibit anemia accompanied by increased tumor formation [30] and our previous studies revealed that HAI-1-deficient *Apc*^Min/+^ mice developed severe anemia compared with control mice [29]. In the present study, blood hemoglobin concentrations were also improved after DHMEQ treatment in HAI-1-deficient *Apc*^Min/+^ mice (Figure 2B). At 12 weeks of age, the number of intestinal tumors was significantly increased in HAI-1-deficient *Apc*^Min/+^ mice (116 ± 8.0, n = 5) compared with control mice (46.7 ± 8.0, n = 6) (Figure 2C). Interestingly, DHMEQ treatment significantly reduced the number of intestinal tumors (63.5 ± 3.0, n = 6) in HAI-1-deficient *Apc*^Min/+^ mice to a level comparable with that in control *Apc*^Min/+^ mice (Figure 2C). On the other hand, the administration of DHMEQ had no effect on body weight gain, hemoglobin concentrations, and tumor formation in control mice (Figure 2A-2C). DHMEQ treatment tended to decrease the sizes of tumors both in HAI-1-deficient *Apc*^Min/+^ mice and control mice. However, the differences were not significant (Figure 2C). Tumor histology was similar between the two groups with or without DHMEQ treatment (Figure 2D). We also examined the effect of DHMEQ on nuclear translocation of RelA/p65 in the tumor tissues of HAI-1-deficient *Apc*^Min/+^ mice. As expected, administration of DHMEQ significantly suppressed the nuclear translocation of RelA/p65 both in tumor and stromal cells of HAI-1-deficient *Apc*^Min/+^ mice (Figure 3A). However, DHMEQ did not affect Wnt signaling, as judged by immunohistochemical analysis of β-catenin (Figure 3B). While nuclear β-catenin-positive tumor cells were modestly increased in the intestinal polyps from HAI-1-deficient *Apc*^Min/+^ mice compared to those from control *Apc*^Min/+^ mice, the nuclear translocation of β-catenin was not alleviated by DHMEQ treatment in both tumors. In addition, the expression level of APC was not altered by the HAI-1 deficiency (Supplementary Figure S2).

## DISCUSSION

In this study, we showed that loss of HAI-1 in the intestine leads to activation of NF-κB signaling. Treatment with DHMEQ, a synthesized small molecule inhibitor of NF-κB, alleviated the HAI-1 loss-induced acceleration of tumor formation in *Apc* mutant mice. Consequently, DHMEQ improved health parameters, such as body weight and hemoglobin concentration, in HAI-1-deficient *Apc*^Min/+^ mice. This study also revealed that DHMEQ has no effect on tumor incidence in control mice. Because it has been reported that activation of NF-κB signaling in intestinal epithelial cells and lamina propria macrophages play a major role in the development of colitis-associated cancer (CAC) in humans [11], this study indicates that DHMEQ or its derivatives may have an implication in CRC prevention and therapy.

Although the precise mechanisms underlying the HAI-1 loss-induced activation of NF-κB signaling remain unclear, some likely mechanisms are suggested based on the proposed function of proteases regulated by HAI-1. One is HGF-mediated activation of NF-κB signaling. HAI-1 is a potent inhibitor of pro-HGF-activating proteases, such as HGF activator, matriptase, hepsin, TMPRSS13, and human airway trypsin-like protease (HAT) [31], and our previous study demonstrated that enhanced HGF activation is observed in tumor tissue and non-tumor mucosa of HAI-1-deficient *Apc*^Min/+^ mice [29]. HGF is known to have important roles in cancer progression through its specific receptor, MET [31]. Furthermore, it has been reported that HGF/MET signaling activates a variety of signaling pathways, such as PI3K/AKT, ERK, NF-κB, and STAT3 [32, 33]. Another candidate for activating NF-κB signaling is uPA. uPA and its receptor uPAR are known to have a tumor-promoting role and are involved in cell processes such as migration, proliferation, and angiogenesis [34]. Previous studies revealed that uPA increases nuclear NF-κB p65 levels in meningioma cells and neutrophils [33, 34]. uPA is produced by cancer cells and stromal cells as a proenzyme uPA (pro-uPA) and is activated in the tumor microenvironment [36]. Similar to pro-HGF, pro-uPA is a substrate for HAI-1-targeted serine proteases, such as matriptase and hepsin [37]. Moreover, expression of uPA was significantly increased both in non-tumor mucosa and tumor tissue in HAI-1-deficient *Apc*^Min/+^ mice (Figure 1C, 1D). Therefore, uPA may have a role in the activation of NF-κB signaling in HAI-1-deficient *Apc*^Min/+^ mice. Protease-activated receptor 2 (PAR-2) signaling may also be involved in NF-κB activation. PAR-2 is expressed by intestinal epithelial cells, and it is well known that trypsin-like serine proteases regulated by HAI-1, such as matriptase and prostasin, and kallikrein-5 (KLK5) activate PAR-2 [38]. PAR-2 activation induces various intracellular signalings, such as p38 mitogen-activated protein kinase [39], Rho [40], and NF-κB signaling [41–43]. A recent study showed that PAR-2 activation by matriptase induces NF-κB activity that leads to the release of pro-tumorigenic inflammatory cytokines in a chemically-induced skin carcinogenesis model [43]. Another evidence is that KLK5-mediated activation of PAR-2 in oral squamous cell carcinoma leads to the activation of NF-κB signaling, which increases expression of inflammatory cytokines and suppresses anti-inflammatory tumor suppressor microRNAs [42]. These studies suggested that in HAI-1-deficient *Apc*^Min/+^ mice intestine, PAR-2 was activated by HAI-1 targeted serine proteases, which may cause the activation of NF-κB signaling. Indeed, the activity of trypsin-like serine protease was significantly increased in HAI-1-deficient *Apc*^Min/+^ mice mucosa (Supplementary Figure S2). While matriptase, a major target of HAI-1 with an efficient PAR-2 activating activity, was expressed in the intestinal mucosa, its mRNA level was not altered by the defect of HAI-1 (Supplementary Figure S2). At present, it remains uncertain whether the increased trypsin-like protease activity in HAI-1-deficient mucosa is caused by dysregulated matriptase or other serine proteases. Taken together, excess activation of HGF, uPA, and/or PAR-2 due to the absence of HAI-1 may induce the activation of NF-κB signaling and produce pro-tumorigenic inflammatory cytokines that may be responsible for increased intestinal tumorigenesis. Further study will be required to clarify the detailed mechanisms underlying the increased intestinal tumorigenesis in HAI-1-deficient *Apc*^Min/+^ mice.

In summary, we demonstrated that treatment with DHMEQ, a small molecule inhibitor of NF-κB, reduced tumor formation and improved the health condition without adverse side effects in HAI-1-deficient *Apc*^Min/+^ mice. This suggests that DHMEQ serves as a promising agent for the treatment of NF-κB-activated colon cancer.

## MATERIALS AND METHODS

### Mice

All animal experiments were approved by the Institutional Animal Care and Use Committee of the University of Miyazaki. HAI-1(*Spint1*)-deficient *Apc*^Min/+^ mice were established by crossing *Spint1*^LoxP/LoxP^ mice with mice harboring the Cre recombinase under the control of villin promoter and *Apc*^Min/+^ mice (*Spint1*^LoxP/LoxP^/Villin-Cre^+/0^/ *Apc*^Min/+^) [29]. *Spint1*^LoxP/LoxP^*Apc*^Min/+^ mice were used as a control.

### Murine intestinal tumorigenesis models

*Apc*^Min/+^ mice were used for analyzing the effect of DHMEQ, a small molecule inhibitor of NF-κB. Eight-week-old male HAI-1/Spint1-deficient and control *Apc*^Min/+^ mice were injected intraperitoneally with 10 mg/kg DHMEQ three times per week and were sacrificed 12 weeks after birth and autopsied to evaluate the number and sizes of tumors formed. Scoring of tumor size was calculated as previously described [29]. For histological analysis, intestinal tissues were fixed in 4% formaldehyde in phosphate-buffered saline (PBS) and embedded in paraffin. Four-micrometer-thick sections were stained with hematoxylin and eosin (HE).

### RT-PCR

Total RNA was prepared with TRIzol™ (Life Technologies Japan, Tokyo, Japan), followed by DNase I (Takara Bio, Shiga, Japan) treatment. For RT-PCR, 3 μg of total RNA was reverse-transcribed with a mixture of Oligo (dT)12-18 (Life Technologies Japan) and random primers (6mers) (Takara Bio) using 200 units of ReverTra Ace™ (TOYOBO, Osaka, Japan), and 1/30^th^ of the resulting cDNA was processed for each PCR reaction with 0.2 μM of both forward and reverse primers and AmpliTaq Gold™ PCR Master Mix (Life Technologies Japan). The thermal cycle profile was 15 sec at 94°C, 15 sec at 60°C, and 1 min at 70°C. The primer sequences for β-actin, TNFα, IL-6, uPA, matriptase and APC are described in Table 1. For qRT-PCR, PCR was performed in a Thermal Cycler Dice Real Time System II (Takara Bio) using the SYBR Premix Ex Taq II (Takara Bio). For internal control, β-actin mRNA was also measured.

**Table 1:** Primer for RT-PCR

Gene	Forward	Reverse	size
β-actin	5′-TGACAGGATGCAGAAGGAGA	5′-GCTGGAAGGTGGACAGTGAG	131
TNF-α	5′-TCGAGTGACAAGCCTGTAGC	5′-GGAGGTTGACTTTCTCCTGG	255
IL-6	5′-AGCCAGAGTCCTTCAGAGAG	5′-ACTCCTTCTGTGACTCCAGC	136
uPA	5′-GAAGTTTGAGGTGGAGCAGC	5′-CCCGTGCTGGTACGTATCTT	101
Matriptase	5′-CACGAATGATGTGTGTGGGTTTC	5′-CCTGGAACATTCGCCCATCT	105
APC	5′-TGTCTGCACACTGCACTGAG	5′-CTTGCTGAGAGATTCCAC	286

### Immunohistochemical analyses

Intestinal tissues of HAI-1-deficient *Apc*^Min/+^ mice and control mice were fixed in 4% paraformaldehyde in PBS overnight and then dehydrated and embedded in paraffin. For immunohistochemistry, tissue sections were processed for antigen retrieval by microwaving for 10 min at 96°C in 10 mM citrate buffer (pH 6.0), followed by treatment with 3% H_2_O_2_ in PBS for 10 min. After blocking in 5% normal goat serum (DAKO, Glostrup, Denmark) in PBS, the sections were incubated with anti-NF-κB p65 rabbit monoclonal antibody (Cell Signaling Technology, Tokyo, Japan) or anti-β-catenin rabbit polyclonal antibody (Sigma-Aldrich, St. Louis, MO) or anti-HAI–1 goat polyclonal antibody (R&D Systems, Minneapolis, MN) for 16 h at 4°C and then incubated with Envision™ labeled polymer reagents (DAKO) for 30 min at room temperature. The reactions were revealed by nickel, cobalt-3, 3′-diaminobenzidine (Pierce, Rockford, IL) and counterstained with Mayer's hematoxylin. To quantify the nuclear translocation of NF-κB or β-catenin, ten randomly selected areas were photographed at 200× magnification in both normal (non-tumor) intestinal mucosal tissue and tumor tissue from each mouse. Then, two independent investigators counted the nuclear RelA or β-catenin-positive cells and the mean number per field were calculated.

### Statistical analysis

Statistical analysis was performed using SPSS15.0 software (SPSS Japan Inc., Tokyo, Japan). Comparison between two unpaired groups was made with the Mann-Whitney U test. Significance was set at *p* < 0.05.

## SUPPLEMENTARY MATERIALS FIGURES


