# Supplementary material for: Inhibition of nuclear factor-κB signaling suppresses Spint1-deletion-induced tumor susceptibility in the ApcMin/+ model
Source: Oncotarget. 2016 Sep 6;7(42):68614–22. doi: 10.18632/oncotarget.11863 (PMC5356577; doi:10.18632/oncotarget.11863)
Supplement: Supplementary file 1 [file oncotarget-07-68614-s001.pdf]

# Inhibition of nuclear factor-κB signaling suppresses *Spint-1*-deletion-induced tumor susceptibility in the *Apc*$^{Min/+}$ model

## SUPPLEMENTARY FIGURES

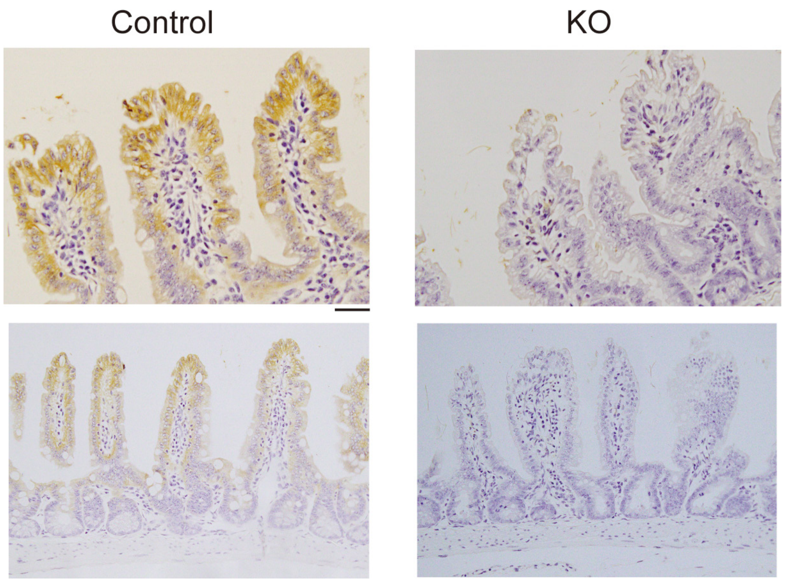


**Supplementary Figure S1: HAI-1 immunostain of control and HAI-1-deficient (KO) *Apc*$^{Min/+}$ mice intestinal tissues.** Bar, 20 (top) and 100 (bottom) µm.

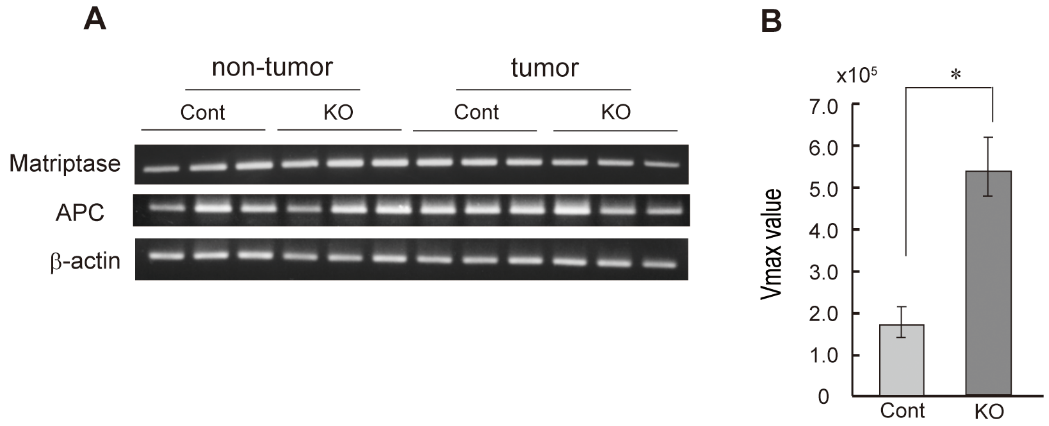


**Supplementary Figure S2: A.** RT-PCR of matriptase and APC in non-tumor mucosa and tumor tissues of control (Cont) and KO mice. **B.** Intestinal non-tumor mucosa of control and KO mice were homogenized and centrifuged. Then, supernatant were collected and used for measurement of protease activity. The fluorogenic substrate, Boc-Glu-Ala-Arg-AMC (peptide institute, Osaka, Japan) was then added to 10uM final concentration, and fluorescence release was measured using FlexStation 3 (Molecular Devices, Tokyo, Japan). *, $p < 0.05$.
